# Near-infrared optical nanothermometry via upconversion of Ho^3+^-sensitized nanoparticles

**DOI:** 10.1038/s41598-023-42034-z

**Published:** 2023-09-08

**Authors:** Sylwia Ryszczyńska, Inocencio R. Martín, Tomasz Grzyb

**Affiliations:** 1 Department of Rare Earths, Faculty of Chemistry, Adam Mickiewicz University, Poznań, Uniwersytetu Poznańskiego 8, 61-614 Poznan, Poland; 2 NanoBioMedical Centre, Adam Mickiewicz University, Poznań, Wszechnicy Piastowskiej 3, 61-614 Poznan, Poland; 3 Departamento de Física, IMN, Universidad de La Laguna, Apdo. 456, 38200 San Cristóbal de La Laguna, Santa Cruz de Tenerife Spain

**Keywords:** Sensors and biosensors, Materials for optics, Lasers, LEDs and light sources, Nonlinear optics, Nanoscale materials, Nanoparticles, Synthesis and processing, Optical spectroscopy

## Abstract

Recently, materials revealing the upconversion (UC) phenomenon, which is a conversion of low-energy photons to higher-energy ones, have attracted considerable attention in luminescence thermometry due to the possibility of precise and remote optical thermal sensing. The most widely studied type of luminescent thermometry uses a ratiometric approach based on changes in the UC luminescence intensity, mainly of lanthanide ions’ thermally coupled energy levels. In this work, NaYF_4_:Ho^3+^@NaYF_4,_ and NaYF_4_:Ho^3+^, Er^3+^@NaYF_4_ nanoparticles (NPs) were synthesized by the controlled reaction in oleic acid and octadecene at 573 K. The obtained nanoparticles had hexagonal structures, oval shapes, and average sizes of 22.5 ± 2.2 nm and 22.2 ± 2.0 nm, respectively. The spectroscopic properties of the products were investigated by measurements of the UC emission under 1151 nm laser excitation in the temperature range between 295 to 378 K. The sample doped with Ho^3+^ and Er^3+^ ions showed unique behavior of enhancing emission intensity with the temperature. The relative sensitivity determined for the NPs containing Ho^3+^ and Er^3+^ ions, reached the maximum value of 1.80%/K at 378 K. Here, we prove that the NaYF_4_:Ho^3+^, Er^3+^@NaYF_4_ system presents unique and excellent optical temperature sensing properties based on the luminescence intensity ratios of the near-infrared bands of both Ho^3+^ and Er^3+^ ions.

## Introduction

The rapid development of technology would be impossible without discoveries and inventions in multiple fields of science. The crucial aspect of various scientific studies is determining the properties of the research objects. The particularly significant parameter is temperature, which is a physical quantity important for all biological processes. Thus, temperature measurements are widely used in medicine, including early tumor detection, monitoring of brain activity, or in vivo inflammation detection^1–5^. In addition, temperature sensors are also relevant in other areas, e.g., for diagnostics of constructions, electrical installations, or even food production^6–9^.

However, it is difficult to verify the actual temperature of the material through a typical contact approach due to the interference from the introduced thermometers^10^. Thus, it is important to find a non-contact system to determine the internal temperature of the object^11–13^. The appealing method for remote temperature detection is luminescence thermometry, based on changes in the emission character of previously excited material^14^. The ability to prepare nanoparticles (NPs), resulting from nanotechnology development, is directly responsible for the remarkable advancements in optical nanothermometry^15^. The NPs’ luminescence highly depends on temperature, so the remote thermal readout is possible through a simple spectroscopic analysis^16^. One of the most widely studied types of optical nanothermometry is the ratiometric approach, which uses changes in the luminescence intensity^17^. However, to measure temperature with this method, the sensor must show at least two emission peaks whose changes in the intensities are interdependent.

The promising candidates for non-contact optical nanothermometry seem to be upconverting nanoparticles (UCNPs)^18^. Upconversion is a process of converting low-energy photons to high-energy ones, usually from the near-infrared (NIR) to the ultraviolet or visible range^19^. This phenomenon occurs for materials doped with lanthanide ions (Ln^3+^), which have a 4f. electronic structure with many well-defined energy levels. The unique properties of the UCNPs containing Ln^3+^ ions result in narrow absorption and emission bands, a significant difference between the absorption and the emission wavelengths, and relatively long luminescence lifetimes^20–23^. The UCNPs can be excited by NIR radiation, which benefits medical applications. Excitation of UCNPs by NIR light results in the low autofluorescence of biological materials, a high signal-to-noise ratio, and negligible photobleaching due to the low-energy excitation inorganic structure of NPs^24^.

Until now, the prevailing types of optical nanothermometers based on UCNPs ions have relied on the temperature-dependent emission from thermally coupled energy levels^25^. The UCNPs with an admixture of Er^3+^ ions are of great interest in this area^3,26^. Researchers frequently employ the thermally-coupled transitions of Er^3+^ ions, specifically from the excited energy levels of ^2^H_11/2_ and ^4^S_3/2_ to the ground energy level of ^4^I_15/2_. Plenty of these studies concern materials doped with Er^3+^ and Yb^3+^ ions, excited by 980 nm radiation, in which the sensitizing Yb^3+^ ions transfer the absorbed energy to the emitting Er^3+^ ions^2,27–29^. However, researchers have also published findings on UCNPs that are solely doped with Er^3+^ and directly excited by radiation at 980 nm or 1500 nm^30,31^. Moreover, a single study reported the temperature sensing properties of Er^3+^ doped NPs, where the luminescence originating from non-thermally coupled bands in the NIR range, specifically ^4^I_9/2_ → ^4^I_15/2_ and ^4^I_11/2_ → ^4^I_15/2_, was utilized for temperature determination^31^. There are also some studies on the optical thermometry of the materials emitting from the thermally or non-thermally coupled states of the other Ln^3+^ ions, e.g., Ho^3+^^32–34^, or Tm^3+^ ions^35,36^, but these materials are in general excited at 980 nm because of co-doping with Yb^3+^ ions.

Using Ln^3+^ ions other than Yb^3+^ in the proper matrix can lead to the UCNPs with properties promising for optical nanothermometry. For instance, a system in which Ho^3+^ ions act as sensitizers and Er^3+^ ions as emission centers can be excited in the NIR range with four different wavelengths (around 755, 900, 1150, and 1950 nm)^20,37,38^. The observed luminescence of these UCNPs displays transition bands originating from both Er^3+^ and Ho^3+^ ions. Depending on the temperature, alterations in the emission intensity of the individual peaks of UCNPs exhibit distinct behavior. This characteristic holds great potential for enhancing temperature sensors.

This work presents a new approach to optical nanothermometry based on the properties of Ho^3+^ and Er^3+^ ions. We selected β-sodium-yttrium fluoride as a matrix for Ln^3+^ ions because β-NaYF_4_ is an excellent UC host material with low phonon energy (around 360 cm^−1^)^39^, reducing the multiphoton quenching processes. In addition, β-NaYF_4_, under the proper synthesis conditions, forms crystalline NPs of small sizes with narrow size distribution. The spectroscopic properties of the prepared NaYF_4_:Ho^3+^@NaYF_4_, and NaYF_4_:Ho^3+^, Er^3+^@NaYF_4_ samples were measured under 1151 nm excitation in the temperature range from 295 to 378 K. The obtained results allowed us to determine the sensitivity of this nanothermometer. Our results show that a different way of excitation than the typical one at 980 nm through Yb^3+^ ions can also result in a good-quality optical thermometry sensor. Moreover, the used approach allows for excitation within the second biological window, which is very convenient for biomedical applications^40^.

## Methods

### Materials

Yttrium, holmium, and erbium chloride hydrates (99.99%, Alfa Aesar), sodium oleate (≥ 82%, Sigma Aldrich), and ammonium fluoride (≥ 98%, Fluka) were used as a source of Y^3+^, Ho^3+^, Er^3+^, Na^+^ and F^-^ ions, respectively. The chlorides were placed in the dryer at 348 K for a week to remove the water (the residual water content was determined by TGA analysis). The reaction was carried out in n-octadecene (90% Alfa Aesar) and oleic acid (70% Fisher Chemicals). Ethanol (99.8% POCh S.A.) and n-hexane (≥ 99% POCh S.A.) were used to purify the post-reaction products.

NaYF_4_:7.5%Ho^3+^,7.5%Er^3+^@NaYF_4_ preparation:

- β-core
  To obtain 5.5 mmol of β-NaYF_4_:7.5%Ho^3+^,7.5%Er NPs, 110 mL of *n*-octadecene and oleic acid mixture (1:1), 4.6750 mmol of yttrium chloride, 0.4125 mmol of both holmium and erbium chlorides were purified at 373 K under vacuum for 2.5 h. 11.0 mmol of sodium oleate (2× excess) and 33.0 mmol of ammonium fluoride (1.5× excess) were separately added to the heated mixture under nitrogen flow and purified at 373 K under vacuum for 30 and 5 min, respectively. The mixture was heated at 573 K with vigorous stirring, under nitrogen flow for 1 h, and cooled down. The post-reaction product was purified five times by sequential dispersing in n-hexane and precipitating by ethanol (5 min, 8000 rpm). The obtained NPs were dispersed in n-hexane and air-dried for 24 h.
- α-shell
  To obtain 15 mmol of α-NaYF_4_ NPs, 300 mL of *n*-octadecene and oleic acid mixture (1:1) and 15 mmol of yttrium chloride were purified at 373 K under vacuum for 3 h. 22.5 mmol of sodium oleate (1.5× excess) and 60.0 mmol of ammonium fluoride were separately added to the heated mixture under nitrogen flow and purified at 373 K under vacuum for 45 and 10 min, respectively. The mixture was heated at 473 K with vigorous stirring, under nitrogen flow for 1 h, and cooled down. The post-reaction mixture was centrifuged (10 min, 9000 rpm), the product was precipitated by adding ethanol and purified three times by sequential disperse in n-hexane and precipitated by ethanol (5 min, 8000 rpm). The obtained NPs were air-dried for 36 h.
- β-core@β-shell
  To obtain β-NaYF_4_:7.5%Ho^3+^,7.5%Er^3+^@β-NaYF_4_ (briefly NaYF_4_:Ho^3+^, Er^3+^@NaYF_4_) NPs, 1 mmol of β-NaYF_4_:7.5%Ho^3+^,7.5%Er^3+^ NPs and 7 mmol of α-NaYF_4_ NPs were added to 32 mL of n-octadecene and oleic acid mixture (1:1) and purified at 373 K under vacuum for 3 h. Then the mixture was heated at 573 K with vigorous stirring, under nitrogen flow for 2 h and 15 min, and cooled down. The post-reaction product was purified four times by sequential dispersing in n-hexane and precipitating by ethanol (5 min, 8000 rpm). The obtained NPs were dispersed in n-hexane or air-dried for 48 h.

*NaYF*_*4*_ *:7.5%Ho* ^3+^ *@NaYF*_*4*_ *preparation:*

The β-NaYF_4_:7.5%Ho^3+^@β-NaYF_4_ (briefly NaYF_4_:Ho^3+^@NaYF_4_) NPs were prepared via the above-mentioned multi-stage procedure under similar synthesis conditions. 5.0875 mmol of yttrium chloride and 0.4125 mmol of holmium chloride were used for the core preparation. The other reagents were added in the same amounts. The previously prepared α-NaYF_4_ NPs were also used for the NaYF_4_:Ho^3+^@NaYF_4_ synthesis.

### Characterization

The purity of the products obtained at individual synthesis stages was determined by Thermogravimetric Analysis (TGA) on Netzsch TG 209 Libra in the temperature range from 298 to 878 K under nitrogen flow (see Fig. S1). The crystalline structures and phase purity of the prepared samples were specified by X-ray Powder Diffraction (XRD) measurements on a Bruker AXS D8 Advance Diffractometer equipped with a Johansson monochromator (λ_Cu_ K_α1_ = 1.5406 Å) and a LynxEye strip detector (step: 0.05° 2θ, step time: 1 s, angular range: 20–100° 2θ). The reference data was taken from JCPDS (00-016-0334). The images of synthesized NPs, based on which the average sizes and size distributions were determined, were recorded on the high-resolution transmission electron microscope Hitachi HT7700 with an accelerating voltage of 120 kV.

Measurements from 295 to 378 K were carried out in a tubular electric furnace (Gero RES-E 230/3), where the sample temperature was controlled via a type K thermocouple in contact with it. The temperature-dependent UC emission spectra of the NaYF_4_:Ho^3+^@NaYF_4,_ and NaYF_4_:Ho^3+^, Er^3+^@NaYF_4_ NPs were obtained using a 10 ns pulsed optical parametric oscillator OPO (EKSPLA/NT342/3/UVE) as the laser source with energy 0.5 mJ. Emissions from the oven were focused on the entrance slit of a spectrograph (Andor SR-303i-A) equipped with a cooled CCD camera (Andor Newton). All spectra were corrected from the spectral response of the equipment. The QuantaMasterTM 40 spectrophotometer equipped with an Opolette 355LD UVDM tunable laser (with a repetition rate of 20 Hz) and a PIXIS:256E digital CCD camera with an SP-2156 imaging spectrograph (Princeton Instruments) was used to measure the dependencies of the energy transitions intensities on the laser energy. The luminescence rise and decay lifetimes were recorded with a Mixed Domain Oscilloscope—200 MHz—Tektronix MDO3022. These measurements were carried out for solid samples at room temperature.

## Results and discussion

### Structural and morphological properties

**Figure 1:**
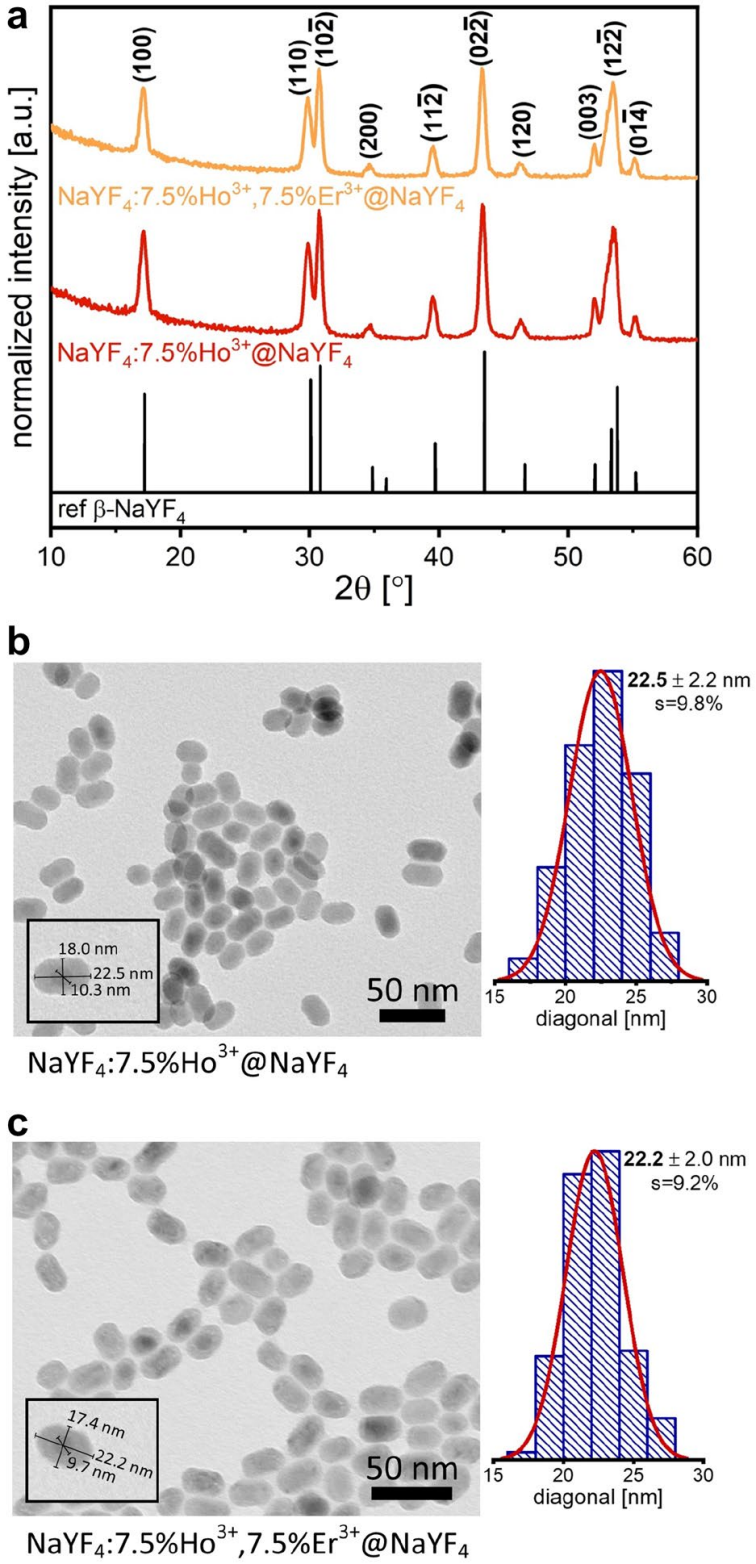
XRD patterns of the NaYF_4_:Ho^3+^@NaYF_4_ and NaYF_4_:Ho^3+^, Er^3+^@NaYF_4_ NPs (**a**), TEM images with corresponding size distributions histograms of the NaYF_4_:Ho^3+^@NaYF_4_ (**b**) and NaYF_4_:Ho^3+^, Er^3+^@NaYF_4_ (**c**) samples.

The prepared NaYF_4_:Ho^3+^@NaYF_4,_ and NaYF_4_:Ho^3+^, Er^3+^@NaYF_4_ NPs crystallized as a single hexagonal phase, with the $\mathrm{P}\overline{6 }$ space group (Fig. 1a). The diffraction peaks of the obtained structures align well with the reference patterns (JCPDS 00-016-0334). No significant shifts in the registered diffractograms were observed because the substitution of Y^3+^ ions with Ho^3+^ or Er^3+^ ions did not affect the unit cell parameters, as all ions are of similar size (${\mathrm{r}}_{{\mathrm{Y}}^{3+}}$ = 1.040 Å, ${\mathrm{r}}_{{\mathrm{Ho}}^{3+}}$ = 1.041 Å, ${\mathrm{r}}_{{\mathrm{Er}}^{3+}}$ = 1.030 Å, see Table S1)^41^. The samples were characterized by small sizes, around 21 nm (by Scherrer equation), as evidenced by broad peaks in the measured diffractograms. The obtained results agree with the TEM images in Fig. 1b,c.

The obtained NPs had oval shapes and average sizes of 22.2/22.5 nm (calculated based on the TEM results) with narrow size distributions (Fig. 1b,c). In the TEM images, a slightly darker region in the centers of the NPs is visible due to about two times higher densities of the Ho^3+^ and Er^3+^ ions added to the core compared to Y^3+^ ions in the shell. Ultimately, the presence of the core@shell structure of the prepared samples was confirmed by the observed increase in their sizes compared with the core-only NPs (see also Fig. S2).

### Spectroscopic properties

**Figure 2:**
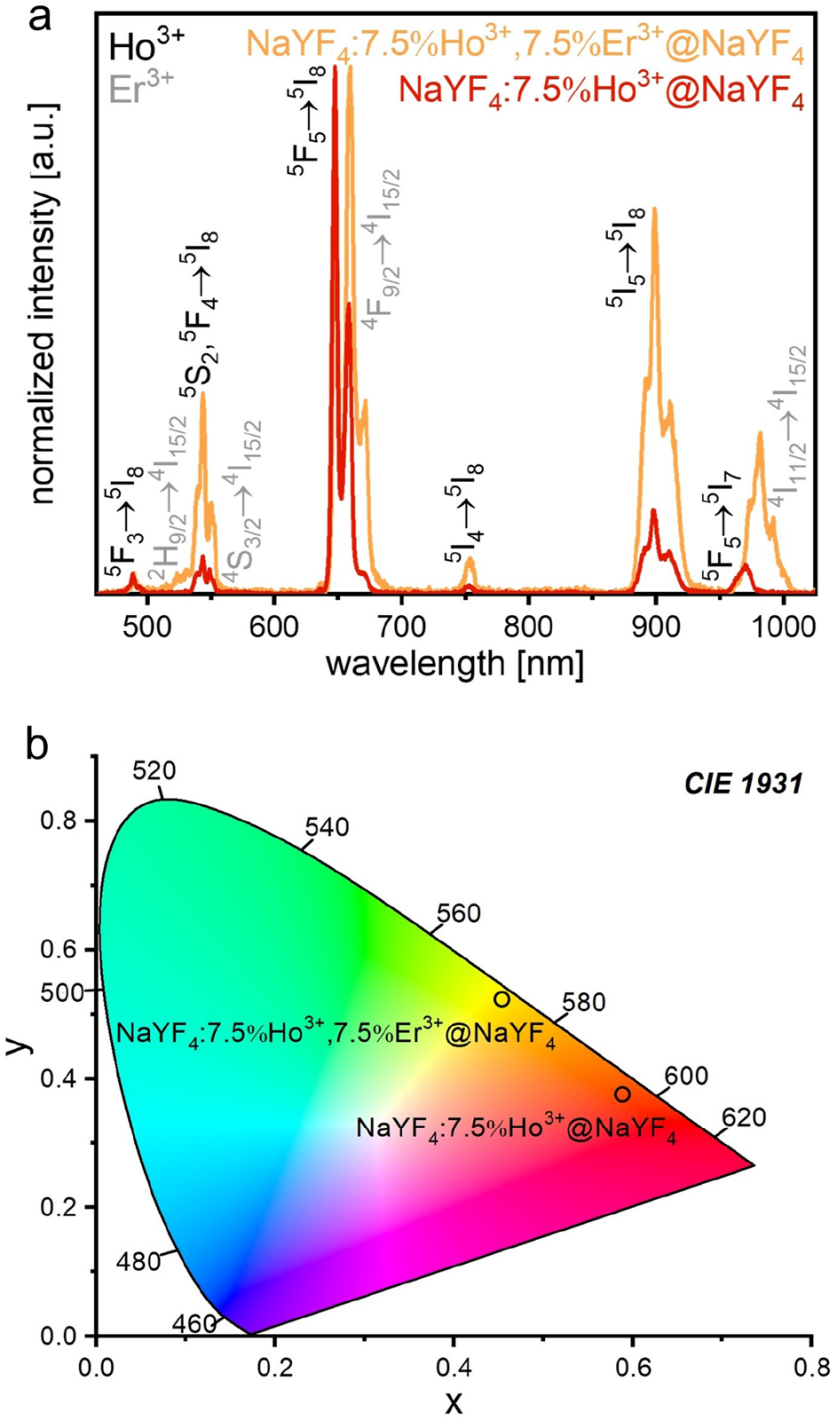
The normalized UC emission spectra of the NaYF_4_:Ho^3+^@NaYF_4_ and NaYF_4_:Ho^3+^, Er^3+^@NaYF_4_ NPs obtained under 1151 nm excitation at room temperature (**a**), CIE chromaticity diagram of prepared samples, prepared based on the corresponding luminescence spectra (**b**).

The spectroscopic studies of the prepared NPs doped with Ho^3+^ only, as well as Ho^3+^ and Er^3+^ ions, revealed inquire and not entirely apparent results. The excitation spectrum (Fig. S3) showed the NIR band in the range from 1130 to 1200 nm, related to the ^5^I_8_ → ^5^I_6_ Ho^3+^ ions energy transition^20^. Based on the recorded spectrum, we selected an 1151 nm laser line to measure the luminescence of the prepared samples. The excitation of the NaYF_4_:Ho^3+^@NaYF_4_ NPs resulted in the UC emission at 489, 544, 648, 752, 898, and 970 nm, connected with the ^5^F_3_ → ^5^I_8_; ^5^S_2_,^5^F_4_ → ^5^I_8_; ^5^F_5_ → ^5^I_8_; ^5^I_4_ → ^5^I_8_; ^5^I_5_ → ^5^I_8_ and ^5^F_5_ → ^5^I_7_ Ho^3+^ ions energy transitions, respectively (Fig. 2a)^20^. The same irradiation of the NaYF_4_:Ho^3+^, Er^3+^@NaYF_4_ NPs revealed the additional luminescence peaks of the Er^3+^ ions. Thus, the bands at around 523, 672, and 982 nm resulted in the ^2^H_9/2_,^4^S_3/2_ → ^4^I_15/2_; ^4^F_9/2_ → ^4^I_15/2_ and ^4^I_11/2_ → ^4^I_15/2_ Er^3+^ ions transitions, respectively, (also Fig. 2a)^20^. The sample containing only Ho^3+^ ions had red emission, while the emission of the Ho^3+^ and Er^3+^ ions doped sample was yellowish-orange (see the CIE chromaticity diagrams in Fig. 2b).

**Figure 3:**
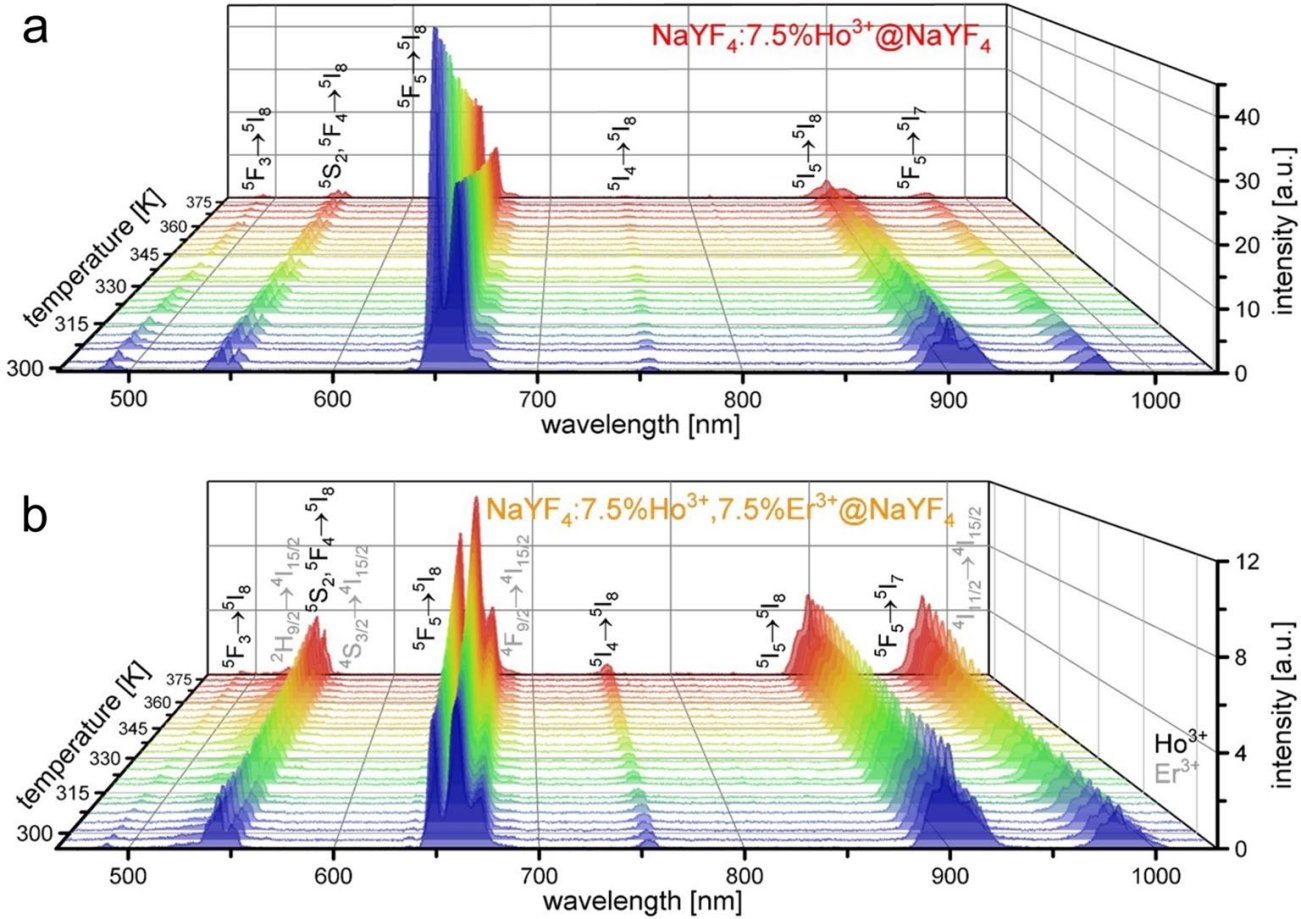
Temperature-dependent UC emission spectra under 1151 nm excitation of NaYF_4_:Ho^3+^@NaYF_4_ (**a**) and NaYF_4_:Ho^3+^, Er^3+^@NaYF_4_ NPs (**b**) NPs.

The UC luminescence spectra of the prepared NPs were measured under 1151 nm excitation in the temperature range from 295 to 378 K (Fig. 3). When the temperature increased, the emission intensity of the NaYF_4_:Ho^3+^@NaYF_4_ sample decreased. Such behavior is consistent with the general tendency of thermal quenching of luminescence due to the intensified non-radiative relaxation processes^11,17^. In contrast, the emission intensity of the NaYF_4_:Ho^3+^, Er^3+^@NaYF_4_ NPs increased with increasing temperature. The luminescence of this sample resulted from certain energy transfers from Ho^3+^ ions to Er^3+^ ions, so the increase of their efficiency with increasing temperature could cause the enhancement of their emission. It is worth noting that the thermal increase of Er^3+^ ions' luminescence intensity, particularly visible at 982 nm, was more significant than the increase of the emission intensity from only Ho^3+^ ions (752, 898 nm).

The parameters characterizing prepared optical temperature sensors were determined using the ratiometric approach. To describe the properties of the NaYF_4_:Ho^3+^@NaYF_4_ sample, the luminescence intensity ratio (*LIR*), which is a ratio of the luminescence intensity from the upper (*I*_*U*_) and lower (*I*_*L*_) states, was used^4,42,43^:

1 $$LIR=\frac{{I}_{U}}{{I}_{L}}$$

The luminescence spectra of the NaYF_4_:Ho^3+^, Er^3+^@NaYF_4_ sample, resulted from the overlapped emission from the energy levels of both Ho^3+^ and Er^3+^ ions. In that case, the *LIR*s were calculated as a ratio of the shorter wavelength peaks’ intensities (*I*_*s*_) to the intensities of the peaks with the longer wavelength (*I*_*l*_):

2 $$LIR=\frac{{I}_{s}}{{I}_{l}}$$

The most important parameter related to temperature-dependent luminescence, especially for the application in optical temperature sensors, is the relative sensitivity (*S*_*R*_) of the material to the temperature changes, determined as the rate of *LIR* changes with the temperature^4,43,44^:

3 $${S}_{R}=\frac{1}{LIR}\frac{d\left(LIR\right)}{dT}100\text{\%}$$

The *S*_*R*_ curves were plotted based on the *LIRs*’ temperature changes of the selected luminescence bands of both Ho^3+^ and Er^3+^ ions (NaYF_4_:Ho^3+^, Er^3+^@NaYF_4_ NPs) or only Ho^3+^ ions (NaYF_4_:Ho^3+^@NaYF_4_ NPs).

**Figure 4:**
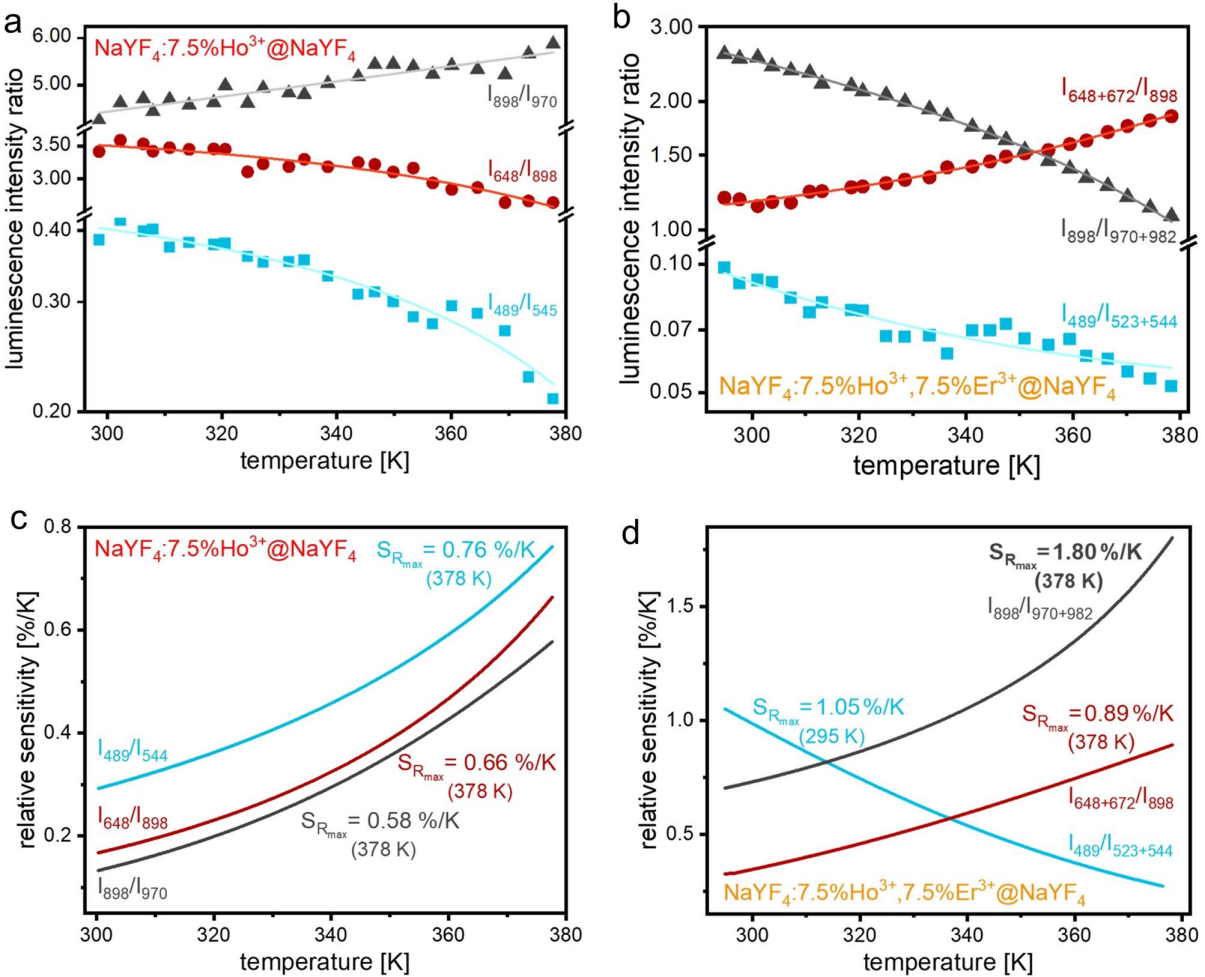
The luminescence intensities ratios (**a**, **b**) and relative sensitivities (**c**, **d**) of the NaYF_4_:Ho^3+^@NaYF_4_ and NaYF_4_:Ho^3+^, Er^3+^@NaYF_4_ NPs.

The *LIR* and *S*_*R*_ dependencies were estimated for the NPs’ luminescence peaks with the different temperature behaviors and are shown in Fig. 4. In the case of the sample doped solely with Ho^3+^ ions, we selected the emission peaks at 489 and 544 nm, 648 and 898 nm, as well as 898 and 970 nm (Fig. 4a,c). When the Ho^3+^ and Er^3+^ ions were dopants, we also took into account the additional peaks that occurred at similar wavelengths: 489 and 523 + 544 nm, 648 + 672 and 898 nm, as well as 898 and 970 + 982 nm (Fig. 4b,d).

The NaYF_4_:Ho^3+^, Er^3+^@NaYF_4_ sample presented relatively high *S*_*R*_ values with a maximum equal to 1.80 (378 K)%/K for the NIR peaks at 898 and 970 + 982 nm. In contrast, all determined *S*_*R*_ values of the NaYF_4_:Ho^3+^@NaYF_4_ sample were below 1.00%/K. The results indicate that the co-doping with Er^3+^ ions to a system based on the Ho^3+^ ions significantly improves the temperature sensing properties, particularly in the NIR range. The prepared NPs exhibit minimum temperature uncertainty around 1.08 K (Fig. S4).

Determining the number of photons involved in populating the excited states of the emitting ions is crucial for explaining the mechanism behind the observed spectroscopic properties of the NPs. The photon's numbers (further described as *n* coefficients) are determined from the dependencies of the luminescence intensities *I*_*UC*_ on the excitation power densities *P*, or in the case of the pulsed laser excitation, from its energies *E*^45:^

4 $${I}_{UC}\propto {P}^{n}\propto {E}^{n}$$

**Figure 5:**
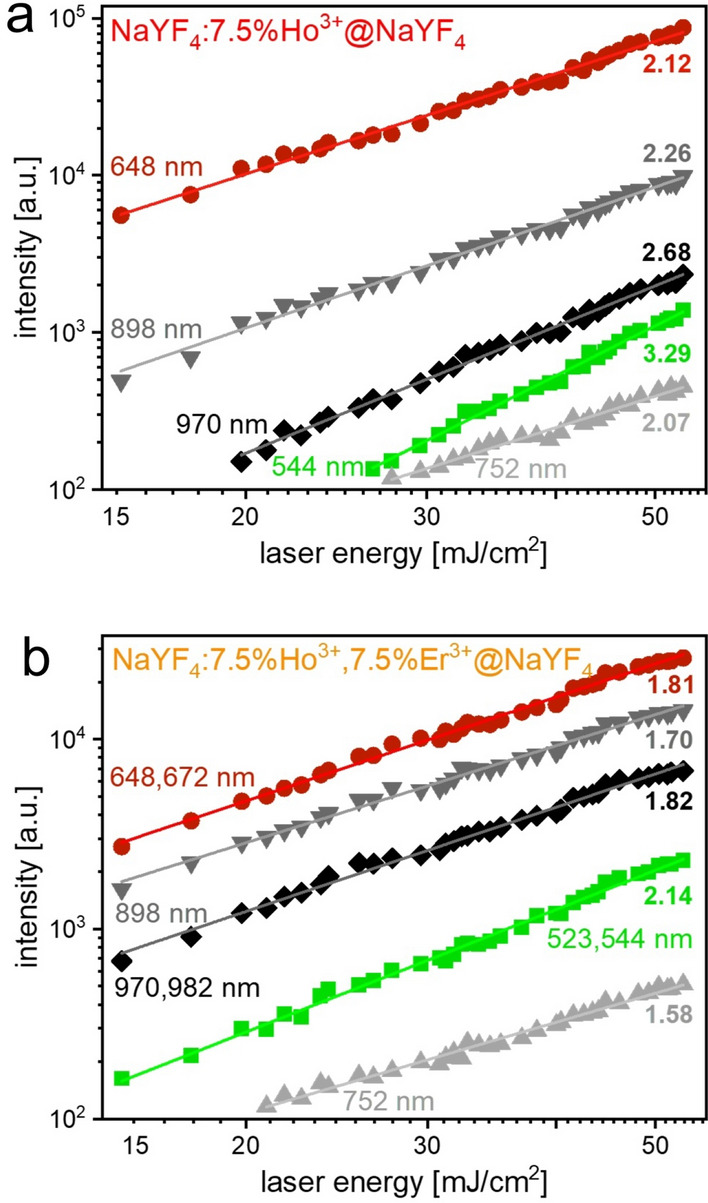
Dependencies of the integral luminescence intensities of the Ho^3+^ or Ho^3+^ and Er^3+^ transition peaks on the laser energy under 1151 nm excitation obtained for the NaYF_4_:Ho^3+^@NaYF_4_ (**a**) and NaYF_4_:Ho^3+^, Er^3+^@NaYF_4_ (**b**) NPs.

Luminescence peaks recorded at similar wavelengths showed significant differences in the *n* coefficient values between both samples (Figs. 5 and S4). The NaYF_4_:Ho^3+^@NaYF_4_ NPs had *n* values mostly between 2.0 and 3.0, which implied that mainly 3 photons are needed to obtain UC emission of Ho^3+^ ions. Only the emission at 544 nm was related to the absorption of 4 photons (*n* = 3.29). The *n* coefficients for the NaYF_4_:Ho^3+^, Er^3+^@NaYF_4_ NPs were mainly below 2.0, suggesting that the observed UC was primarily influenced by processes that necessitated the absorption of only 2 photons. The exception was green emission, which resulted from 3 photons process (*n* = 2.14).

**Figure 6:**
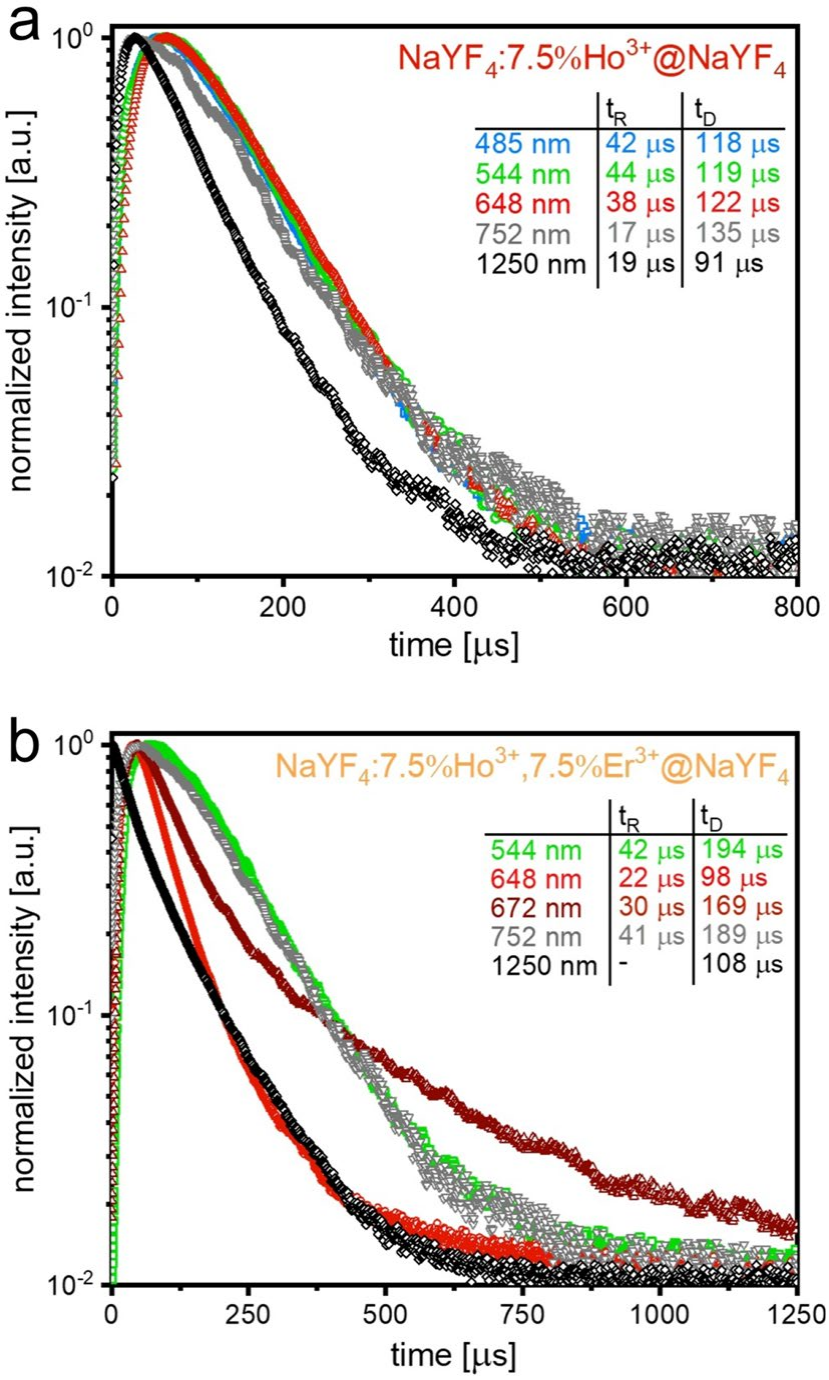
Luminescence lifetime profiles of the NaYF_4_:Ho^3+^@NaYF_4_ (**a**) and NaYF_4_:Ho^3+^, Er^3+^@NaYF_4_ (**b**) NPs obtained under 1151 nm excitation (insets contained estimated rise and decay times).

To fully understand the nature of the observed UC phenomenon, we measured the luminescence lifetimes of the selected Ho^3+^ and Er^3+^ transitions (Fig. 6) and used the following equations to calculate average (effective) rise and decay times^46^:

5 $${{t}_{\mathrm{eff}}}_{R}= \frac{\underset{0}{\overset{{t}_{p}}{\int }}tI\left(t\right)\mathrm{d}t}{\underset{0}{\overset{{t}_{p}}{\int }}I\left(t\right)\mathrm{d}t}$$

6 $${{t}_{\mathrm{eff}}}_{D}= \frac{\underset{{t}_{p}}{\overset{\infty }{\int }}tI\left(t\right)\mathrm{d}t}{\underset{{t}_{p}}{\overset{\infty }{\int }}I\left(t\right)\mathrm{d}t}$$

where *t*_*eff*_ is the effective rise (*R*) or decay (*D*) time, *t*_*p*_ is the time when the lifetime trend changes from rise to decay and *I* is the intensity at time *t* (see insets in Fig. 6).

The UC luminescence of the NaYF_4_:Ho^3+^@NaYF_4_ NPs results from energy transfer (ET) processes between Ho^3+^ ions, as evidenced by the visible rise times of the registered transitions (Fig. 6a). Moreover, the similar rise times of the transitions responsible for the 1250 and 752 nm emission (*t*_*D*_ values around 20 μs) indicate that the ^5^I_6_ and ^5^I_4_ Ho^3+^ levels are likewise populated. The ^5^F_5_, ^5^S_2,_ and ^5^F_3_ states related to the emission at 648, 544, and 489 nm are also populated similarly, slower than the previously mentioned, as evidenced by the congruous rise times close to 40 μs. In the case of the NaYF_4_:Ho^3+^, Er^3+^@NaYF_4_ sample, the UC luminescence at 1250 nm results from quick processes since the rise time of the ^5^I_6_ → ^5^I_8_ transition is not visible. The other emissions, which have rise times below 20 μs, are connected with the energy transfer processes between Ho^3+^ and Er^3+^ ions. In the recorded lifetime profiles of the Ho^3+^ ion transitions, the Er^3+^ ion influence is visible, especially for the red emission (see Fig. 6b).

**Figure 7:**
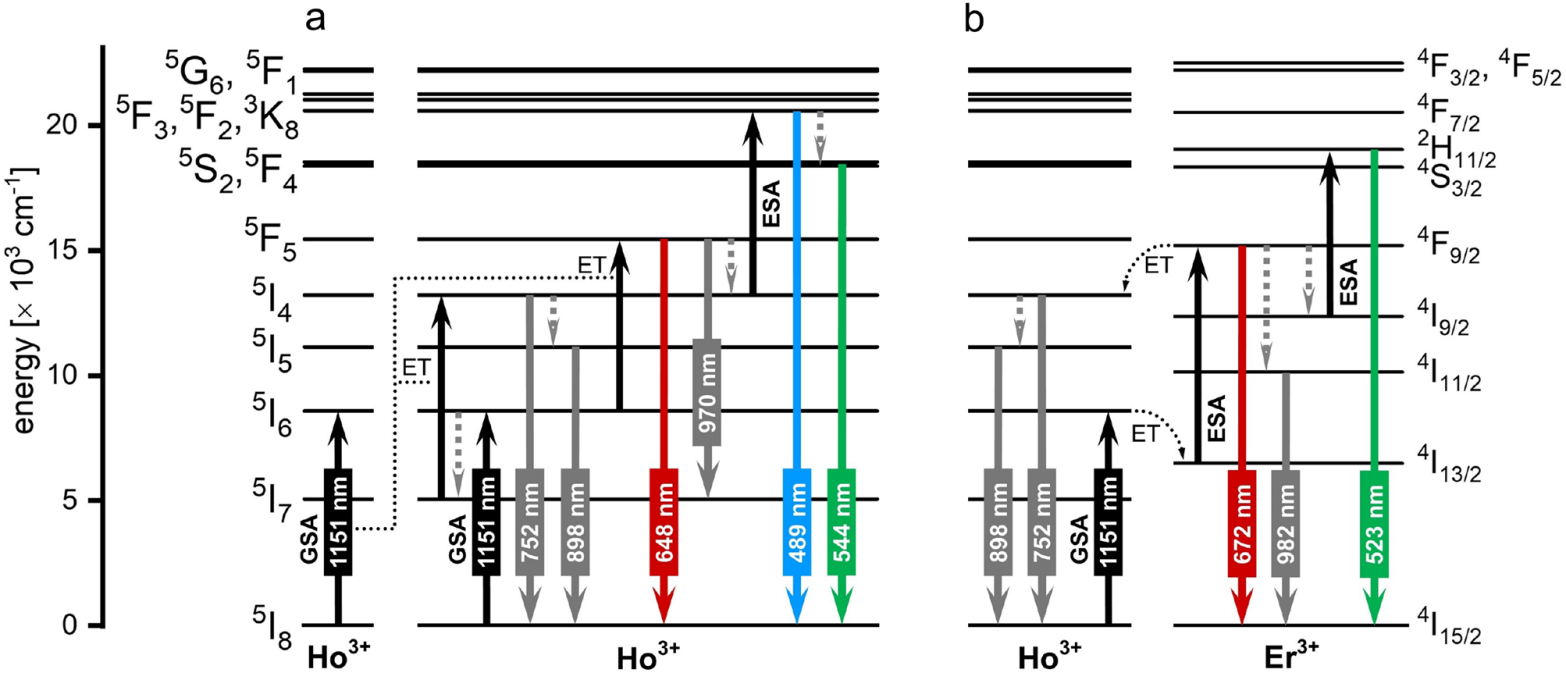
The proposed mechanism of the NaYF_4_:Ho^3+^@NaYF_4_ (**a**) and NaYF_4_:Ho^3+^, Er^3+^@NaYF_4_ (**b**) NPs observed under 1151 nm excitation^49–51^.

The results of the spectroscopic measurements of the prepared NPs became the basis for the proposed mechanism responsible for the observed UC emission under 1151 nm excitation (Fig. 7).

The irradiation of the NaYF_4_:Ho^3+^@NaYF_4_ NPs with the 1151 nm pulsed laser produces Ho^3+^ ions in their ^5^I_6_ excited state via ground state absorption (GSA) process (Fig. 7a). The initially excited Ho^3+^ ions exchange energy with each other by the ET processes. The absorption of the subsequent photon leads to the population of the Ho^3+^ ions into the ^5^I_4_ levels and the weak emission at 752 nm. From this state, there is also relaxation to the ^5^I_5_ state, from which the emission at 898 nm occurs. Simultaneously, the absorption of another photon by the ET process produces the Ho^3+^ ions in their ^5^F_5_ state. The emission at 648 nm (^5^F_5_ → ^5^I_8_) and 970 nm (^5^F_5_ → ^5^I_7_) is from this energy level. The Ho^3+^ ions, previously populated to the ^5^I_4_ level, absorb another photon by the quick ESA process, whereby the Ho^3+^ ions are excited to their ^5^F_3_ energy levels. Hence, the sample presented emissions at 489 nm and after relaxation at 544 nm.

In the case of the NaYF_4_:Ho^3+^, Er^3+^@NaYF_4_ NPs, most of the energy absorbed by Ho^3+^ ions is transferred to Er^3+^ ions (see Fig. 7b). The Er^3+^ ions are excited to their ^4^F_9/2_ levels from where an additional emission is possible giving a peak at 672 nm. Further, intense emission at 982 nm occurs after the relaxation to the ^4^I_11/2_ state. Another photon absorbed within the Er^3+^ ion leads to a population of the ^2^H_11/2_, ^4^S_3/2_ Er^3+^ ions levels and an additional emission band at 523 nm. The emission of Ho^3+^ ions at 890 and 743 nm results from energy back transfer from the excited Er^3+^ ions and is weaker than in the case of a system containing only Ho^3+^ ions^47,48^. The emission of Ho^3+^ ions in NaYF_4_:Ho^3+^, Er^3+^@NaYF_4_ sample probably also consists of the processes occurring in sample NaYF_4_:Ho^3+^@NaYF_4_ sample, however, they are less intense because the pre-excited Ho^3+^ ions transferred most of the energy to Er^3+^ ions.

## Conclusions

Using the precipitation reaction in the oleic acid/octadecene solution, we successfully obtained core@shell UCNPs based on sodium yttrium fluorides doped with either Ho^3+^ ions or both Ho^3+^ and Er^3+^ ions. The prepared UCNPs exhibited an oval shape and average sizes of approximately 22.5 nm.

The NaYF_4_:Ho^3+^@NaYF_4_ and NaYF_4_:Ho^3+^, Er^3+^@NaYF_4_ samples showed UC emission under 1151 nm pulsed laser excitation. We registered the emission of the products in the 295 to 378 K temperature range to determine their temperature-sensing properties.

The NPs containing Ho^3+^ and Er^3+^ ions revealed unusual behavior manifested by increased luminescence intensity with the temperature increase. This observation can be attributed to the specificity of the UC mechanism based on energy transfers from Ho^3+^ to Er^3+^ ions. Upon the research, we discovered that the NaYF_4_:Ho^3+^, Er^3+^@NaYF_4_ NPs have great potential as a temperature sensor based on the excitation and emission in the range of biological windows. This sample shows intense NIR luminescence from Ho^3+^ ions at 899 and 970 nm and Er^3+^ ions at 982 nm. The relative sensitivity determined for these peaks reached the maximum value of 1.80%/K at 378 K. This optical temperature sensor based on the NIR UC emission of the system containing Ho^3+^ and Er^3+^ ions has been reported for the first time. The possibility of excitation within the second biological window and detecting temperature changes in emission intensity around the first biological window make our UCNPs promising candidates for biomedical applications. However, the obtained UCNPs presented high sensitivities not only in the NIR range, which generally makes them excellent candidates for temperature sensing applications not only limited to biological ones.

### Supplementary Information

Supplementary Information.

## Data Availability

The datasets used and/or analyzed during the current study available from the corresponding author on reasonable request.
