# Supplementary material for: Near-infrared optical nanothermometry via upconversion of Ho3+-sensitized nanoparticles
Source: Sci Rep. 2023 Sep 8;13:14819. doi: 10.1038/s41598-023-42034-z (PMC10491596; doi:10.1038/s41598-023-42034-z)
Supplement: Supplementary file 1 — Supplementary Information. [file 41598_2023_42034_MOESM1_ESM.docx]

**Supporting Information**

**for**

**Near-Infrared Optical Nanothermometry via Upconversion of Ho^3+^-Sensitized Nanoparticles**

Sylwia Ryszczyńska^1,2^, Inocencio R. Martín^3^, Tomasz Grzyb^1*^

*^1^Department of Rare Earths, Faculty of Chemistry,*

*Adam Mickiewicz University in Poznań, Uniwersytetu Poznańskiego 8, 61-614 Poznań, Poland*

*^2^NanoBioMedical Centre,*

*Adam Mickiewicz University in Poznań, Wszechnicy Piastowskiej 3, 61-614 Poznań, Poland*

*^3^Departamento de Física, Universidad de La Laguna, Apdo. 456. E-38200 San Cristóbal de La Laguna, Santa Cruz de Tenerife, Spain*

e-mail: tgrzyb@amu.edu.pl


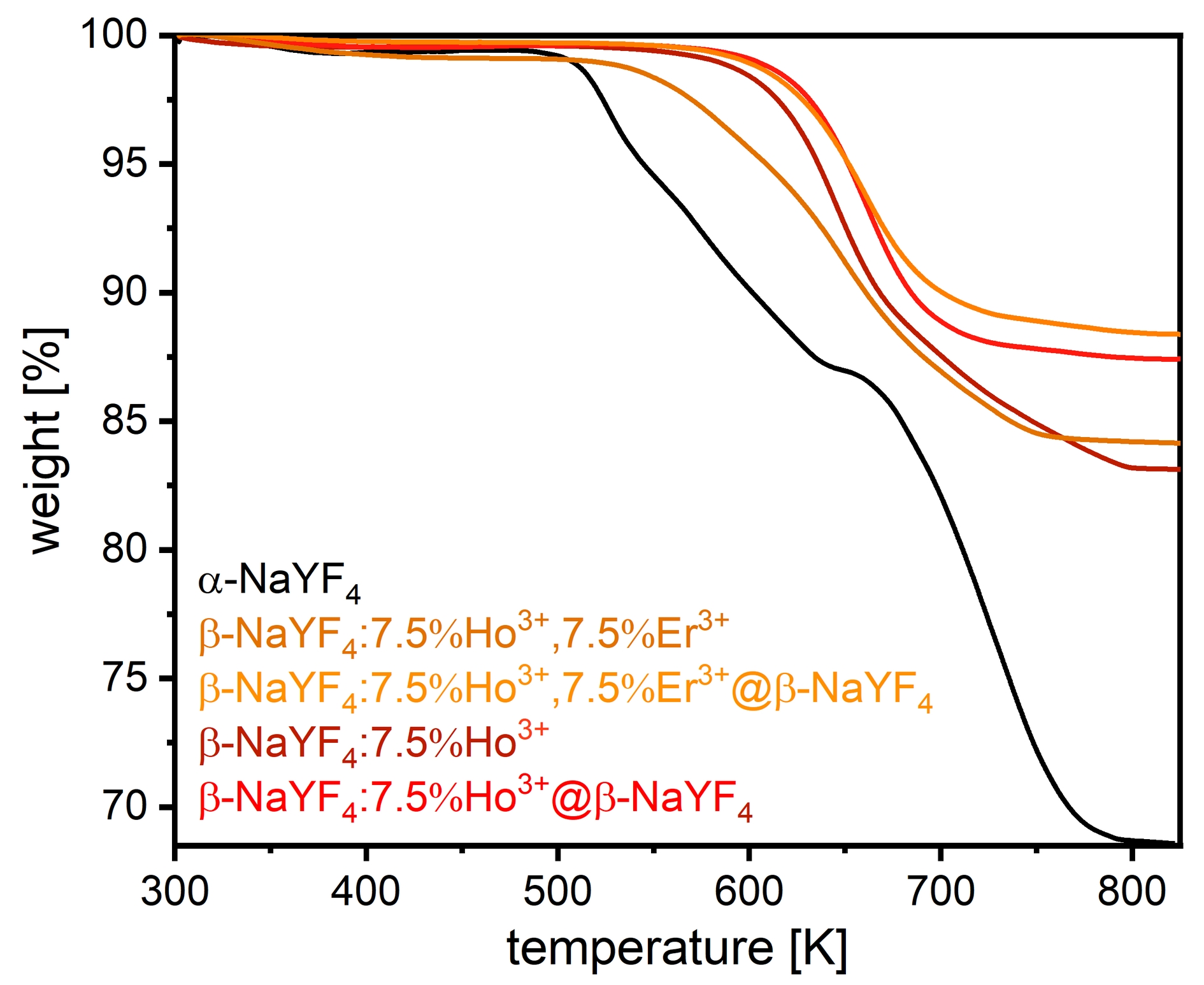


**Fig. S1.** TGA curves of the products obtained at individual synthesis stages.

**Table S1.** Unit cell parameters for cubic and hexagonal NPs structures (calculated by Maud software version: 2.94).^1^

| sample | a [Å] | c [Å] | V [Å^3^] | NPs size calculated based on Scherrer’s equation^2^ [nm] |
| --- | --- | --- | --- | --- |
| β-NaYF_4_ (DOE Data Explorer mp-37267) | 6.071 | 7.105 | 226.81 | – |
| β-NaYF_4_:7.5%Ho^3+^@β-NaYF_4_ | 6.002 | 7.045 | 219.80 | 20.9 |
| β-NaYF_4_:7.5%Ho^3+^,7.5%Er^3+^@β-NaYF_4_ | 5.985 | 7.043 | 218.50 | 20.6 |


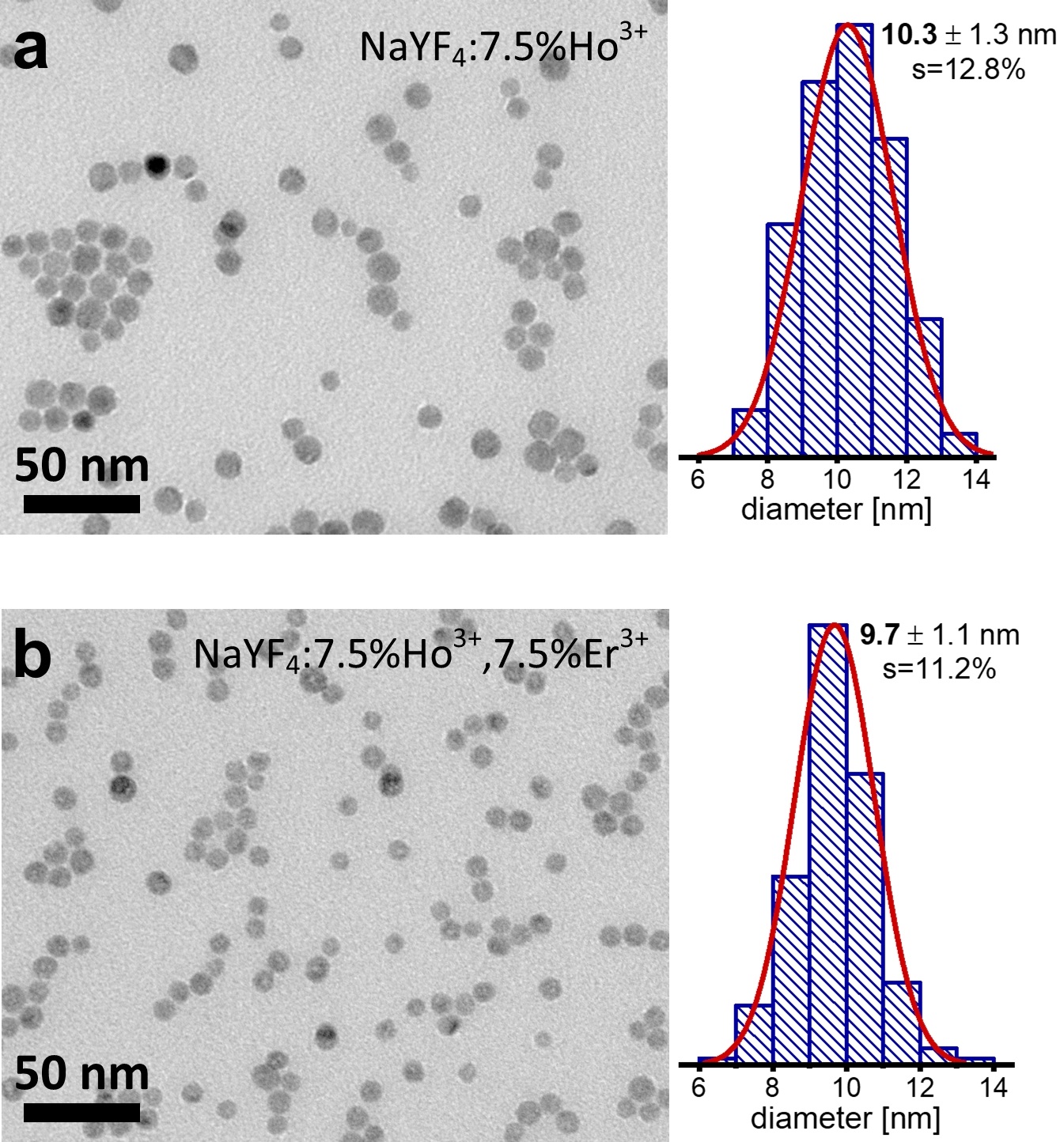


**Fig. S2.** TEM images with corresponding size distributions histograms of the core-only NaYF_4_:Ho^3+^ (a)
and NaYF_4_:Ho^3+^,Er^3+^ (b) NPs.


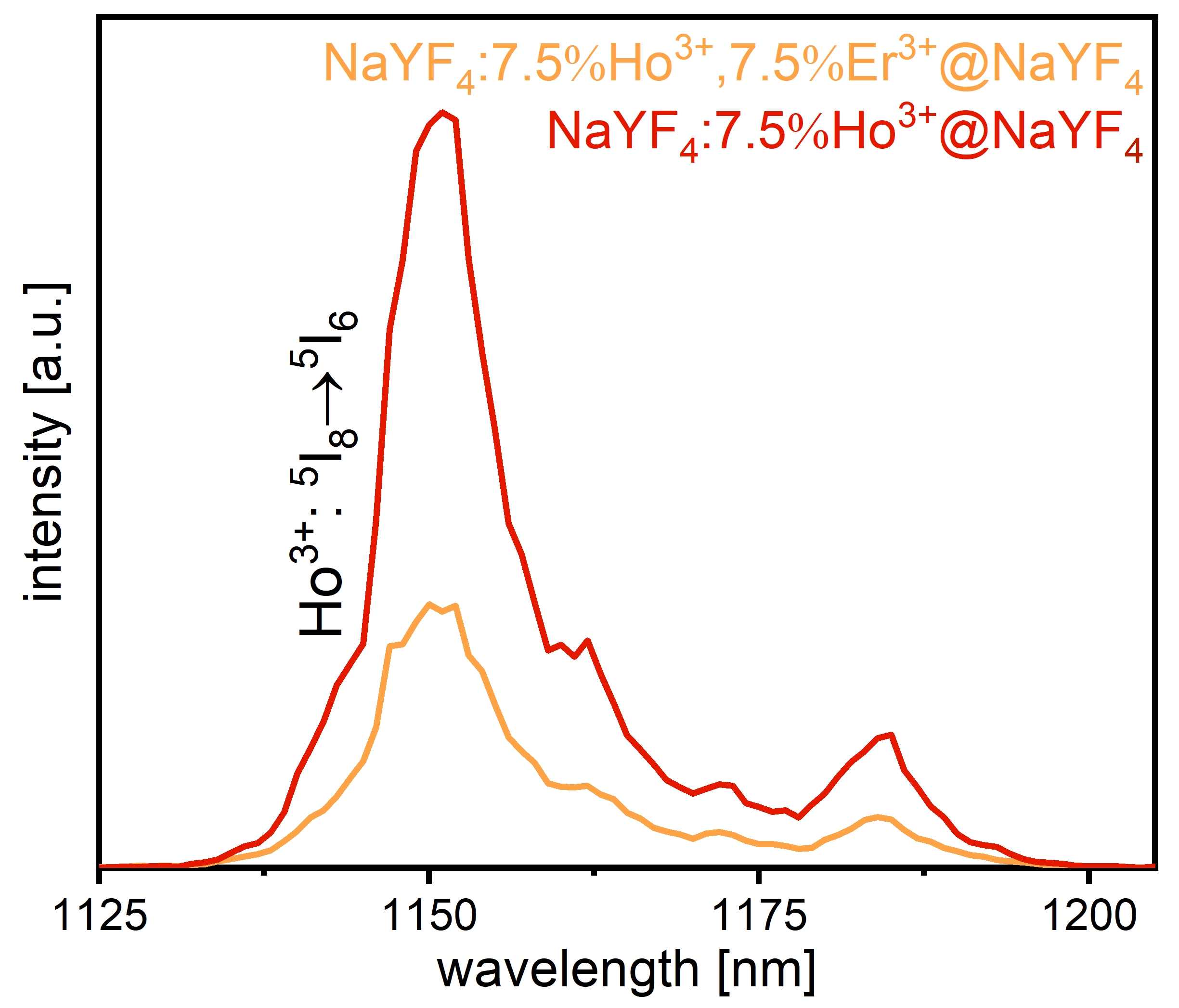


**Fig. S3.** The excitation spectrum of the NaYF_4_:Ho^3+^@NaYF_4_ and NaYF_4_:Ho^3+^,Er^3+^@NaYF_4_ samples (recorded with the observation of the 648 nm luminescence band).


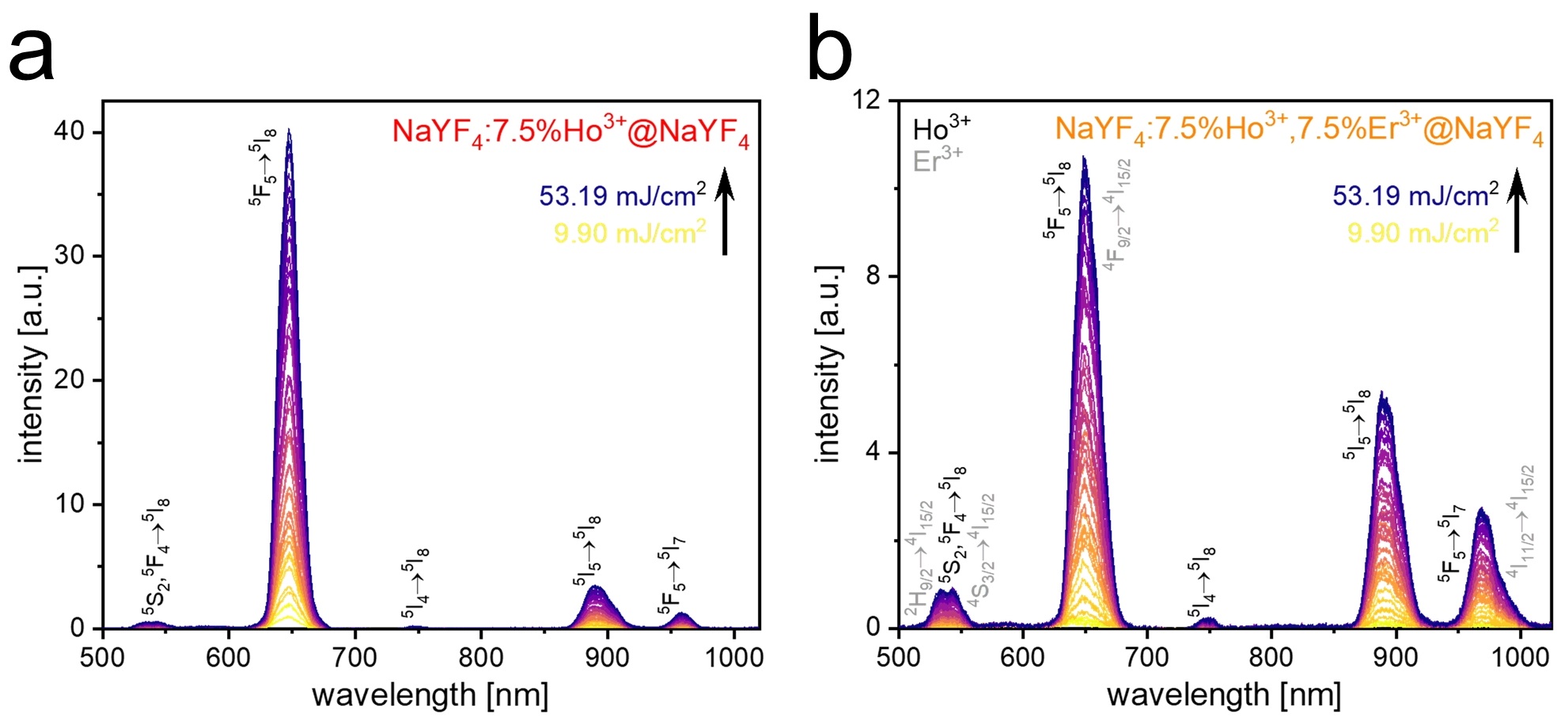


**Fig. S4.** The UC luminescence spectra of the NaYF_4_:Ho^3+^@NaYF_4_ (a) and NaYF_4_:Ho^3+^,Er^3+^@NaYF_4_ (b) NPs obtained under 1151 nm pulsed laser excitation with the energies from 9.90 to 53.19 mJ/cm^2^.


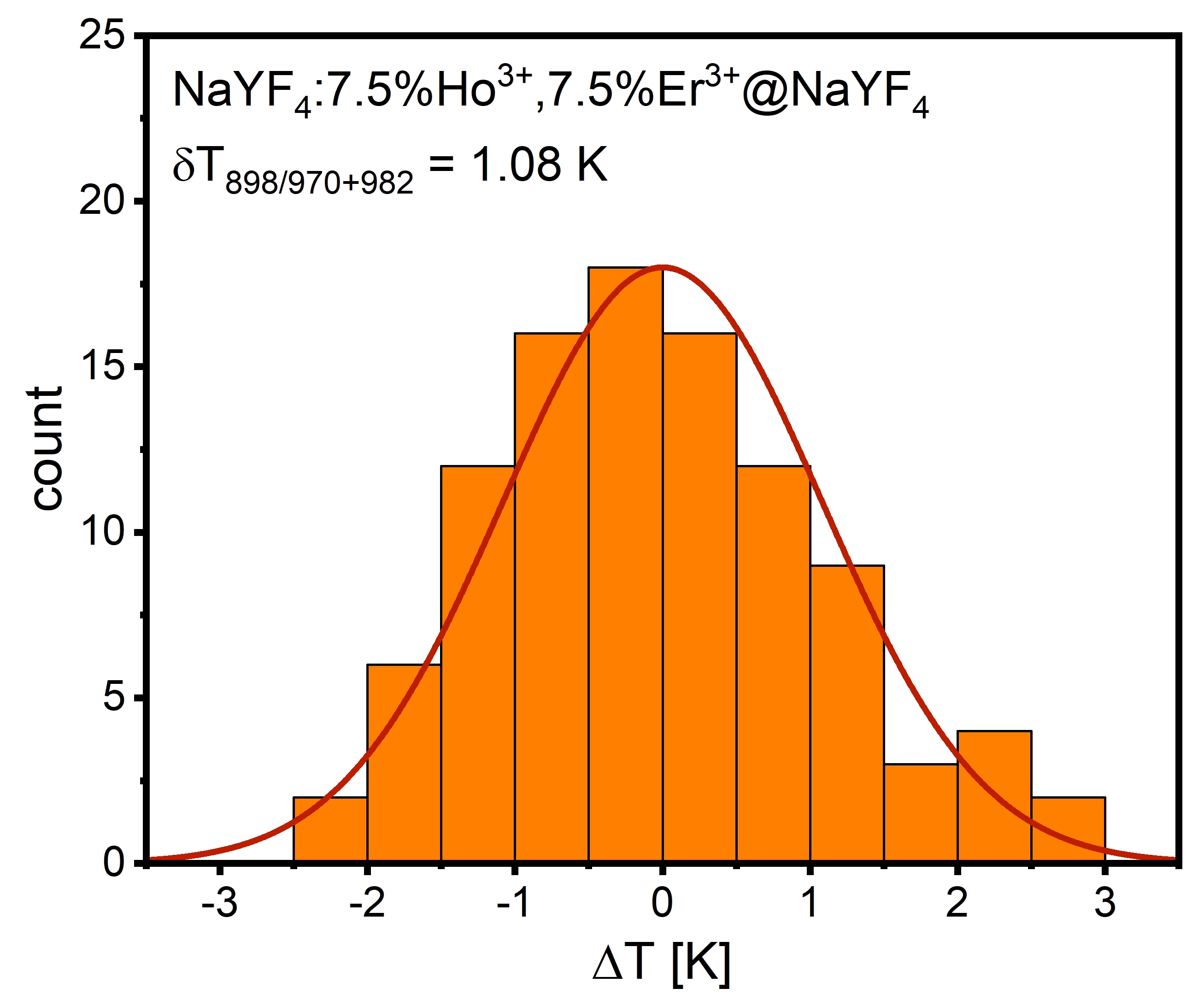


**Fig. S5.** Temperature uncertainty determined from 100 measurements of emission at room temperature for NaYF_4_:Ho^3+^,Er^3+^@NaYF_4_ sample under 1151 nm excitation, based on the 898 to 970+982 nm LIR.

**References**

1. Lutterotti, L. Total pattern fitting for the combined size–strain–stress–texture determination in thin film diffraction. *Nucl. Instruments Methods Phys. Res. Sect. B Beam Interact. with Mater. Atoms* **268**, 334–340 (2010).

2. Patterson, A. The Diffraction of X-Rays by Small Crystalline Particles. *Phys. Rev.* **56**, 972–977 (1939).
